# The complete mitochondrial genome of *Rhinogobius szechuanensis* (Gobiiformes: Ggobiidae: Gobionellinae)

**DOI:** 10.1080/23802359.2023.2167473

**Published:** 2023-02-02

**Authors:** Wen Zhao Liu, Lin Song, Xiao Jiang Chen, Hai Xia Liu

**Affiliations:** a College of Fisheries and Life Science, Dalian Ocean University, Dalian City, Liaoning Province, P.R. China; b College of Fisheries Science and Technology, Jiangsu Agri-animal Husbandry Vocational College, Taizhou, Jiangsu Province, P.R. China

**Keywords:** Mitochondrial genome, *Rhinogobius szechuanensis*, Gobionellinae, freshwater goby, *Rhinogobius rubromaculatus*

## Abstract

Here, we sequenced the complete mitogenome of *Rhinogobius szechuanensis* using the Illumina HiSeq platform and submitted the genome to Genbank with accession number OM617727. Assembly circular mitogenome (16,492 bp) consisted of 54.4% AT content, 13 protein-coding genes, 22 tRNA genes, two rRNA genes, an origin of light-strand replication, and a control region. Phylogenetic analysis supported that *R. szechuanensis* was grouped with *R. rubromaculatus* and clustered with other *Rhinogobius* species. The basal molecular data will be essential for further genetics studies such as evolution, taxonomy, DNA barcoding, and population genetics of *Rhinogobius.*

## 1. Introduction

**Figure 1.**
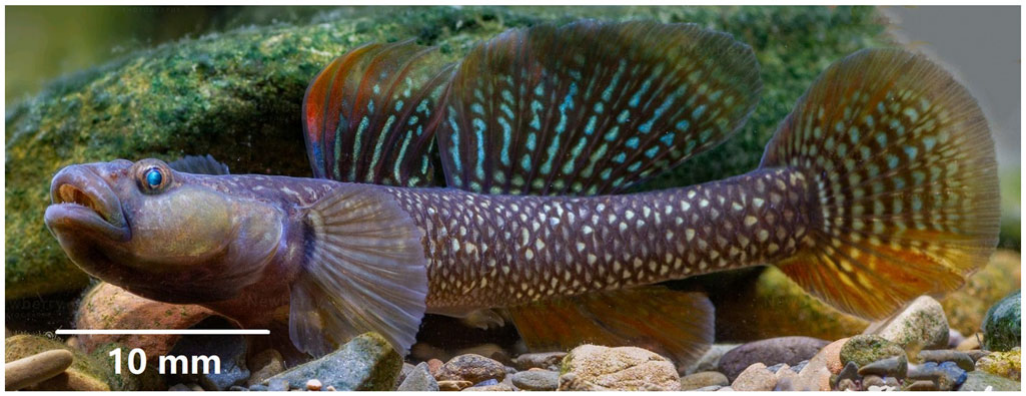
*Rhinogobius szechuanensis*. Specimen was taken from the Minjiang River, Dujiangyan City, Sichuan Province, China. Photographs by Lin Song on 20 June 2021.

*Rhinogobius* (Gill, 1859) is a genus widely distributed in the Western Pacific and East Asia, with approximately 129 species according to Fishbase statistics, and shows high species abundance and ecological diversity (Huang et al. 2016). In recent years, great advances have been made in resolving the phylogeny of *Rhinogobius. Rhinogobius szechuanensis* (Tchang, 1939), which belongs to the genus *Rhinogobius* within the subfamily Gobionellinae, is a freshwater goby endemic to the Minjiang River drainage of China. It is a warm temperate small-scale bottom fish, often inhabiting in flowing streams and rivers, and mostly moving among rocks and pebbles, and it feeds on small fish, aquatic insects, shrimps and crabs (Wu and Zhong, 2008). Because of its small size (∼ 70 mm) and unique body color, it is an ornamental fish popular in aquaria. It can be recognized by dorsal fin VI, I-8-9; anal fin I-7-8; pectoral fins 16–17; abdominal fin I-5; caudal fin 2 + 16 + 4; longitudinal scales 32–34; transverse scales 10–11; dorsal fin anterior scale 0; gill rake 9; no sensory canal; brown margin of each scale on the side of the body, forming reticular patterns (Wu and Zhong 2008) (Figure 1).

*Rhinogobius szechuanensis* has long been classified into the genus *Glossogobius* within the subfamily Gobiinae in the past based on morphological characteristics (Zhang 1996; Ye et al. 2015). Later, researchers proposed to classify it into the genus *Rhinogobius* within the subfamily Gobionellinae (Chen et al. 2008; Huang et al. 2016; Zhang and Zhao 2016). Up to present, only the *12S rRNA* of *R. szechuanensis* was sequenced by Miya et al. (2020), and still lacks strong molecular data to support whether it belongs to *Rhinogobius*. This research aimed to achieve the complete mitochondrial genome of *R. szechuanensis* to clarify its phylogenetic position. The mitogenome is useful as a reference for molecular species identification, and further genetics studies. Therefore, the research is of great significance.

## 2. Materials and methods

### 2.1. Sample collection and preservation

In this study, animals were collected under the permission of Jiangsu Agri-animal Husbandry Vocational College (NSF2021ZR14). Samples of *R. szechuanensis* were obtained from the Minjiang River, Dujiangyan City, Sichuan Province of China (31.0189° N, 103.5879° E) in June 2021. According to the morphological characteristics of *R. szechuanensis* provided by Wu and Zhang, (2008), the fish were identified by means of anatomical microscope and other tools. The samples were quickly frozen with liquid nitrogen and stored at −80 °C, and then transferred to the laboratory. The specimen was preserved in Aquatic Science and Technology Institution Herbarium (https://www.jsahvc.edu.cn/; Voucher number ASTIH-21b1108d26, Chen Xiao Jiang, 2007020030@jsahvc.edu.cn).

### 2.2. DNA extraction and sequencing

The total genomic DNA was extracted from a specimen muscle using the Tguide Cell/tissue genomic DNA Extraction Kit (OSR-M401) (Tiangen, Beijing, China). DNA samples were subjected to quality control: a) The concentration and purity of the samples were determined using NanoDrop 2000 (Thermo Fisher Scientific, USA), which required concentrations ≥20 ng/µL, total amount ≥ 100 ng, and OD_260_/OD_280_ = 1.8–2.2; b) The sample integrity was checked by agarose gel electrophoresis, which required that a major band of genomic DNA be visible and that no appreciable diffusion of degradation exists. The sequencing library was prepared by random fragmentation of the DNA sample, followed by PCR amplification, size selection and library quality check. The DNA raw reads were obtained from the sequencing of the constructed library on Illumina HiSeq 4000 Sequencing platform (Illumina, CA, USA). After the quality check process was conducted on FastQC Version 0.11.8 (Andrews 2015), the filtered reads were assembled into the complete mitogenome using MetaSPAdes with default parameters (Nurk et al. 2017), and the preliminary assembly results including maximum depth, minimum depth and average depth were 33,179 bp, 500 bp and 769.89 bp, respectively.

The resulting circular contig consensus sequence was annotated and verified with MITOS WebServer (http://mitos.bioinf.uni-leipzig.de/index.py) (Bernt et al. 2013). Organelle Genome Maps was drawn by OGDRAW (https://chlorobox.mpimp-golm.mpg.de/OGDraw.html) (Greiner et al. 2019).

### 2.3. Phylogenetic analysis method

MEGA X was used to conduct the molecular phylogenetic (Kumar et al. 2018), and Maximum-likelihood (ML) method was applied to perform the phylogenetic analysis with 1000 bootstrap replicates. The phylogenetic tree based on 13 PCGs of *R. szechuanensis* and 22 published species of Gobionellinae, with *Mugilogobius abei* as outgroup. The best evolutionary model of the concatenated sequences was simulated to be mtREV + G + I + F for the lowest Bayesian information standard scores.

## 3. Results and discussion

### 3.1. Characteristics of R.s szechuanensis mitochondrial genome

**Figure 2.**
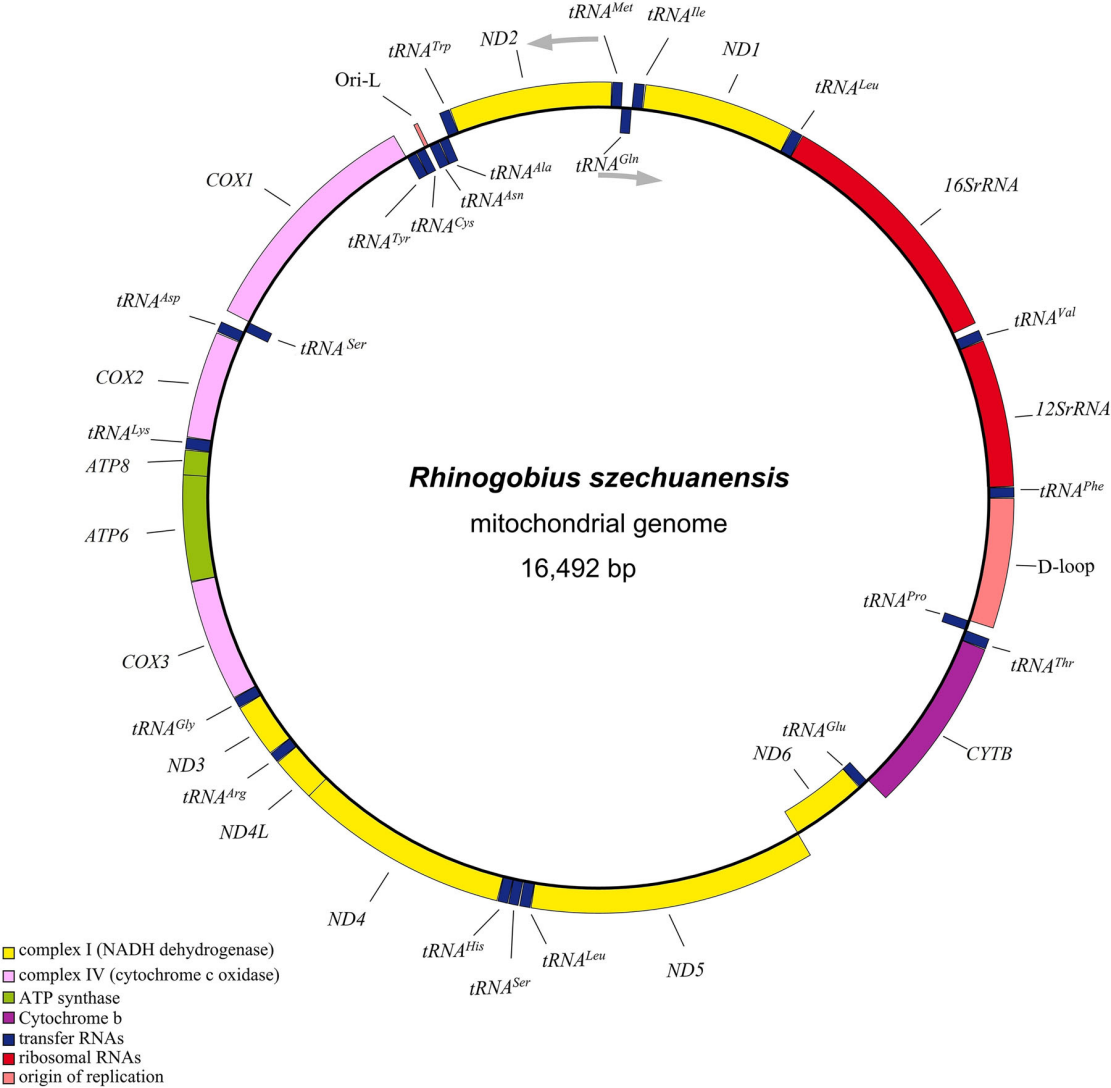
The complete mitochondrial genome map of *R. szechuanensis* (GenBank accession no. OM617727), consisting of 13 PCGs, 22 tRNAs, two rRNAs, the arrows represent direction of transcription. Genes encoded on H-strand and L-strand are displayed outside and inside the ring, respectively.

The *R. szechuanensis* circular 16,492 bp mitogenome was composed of the following nucleotide base composition: 27.4% A, 27% T, 16.4% G, and 29.1% C, which demonstrated the AT-rich (54.4%) feature. The genome organization and transcriptional direction were identical to those of all known goby mitogenomes (Xie et al. 2015; Wang et al. 2019), containing 13 protein-coding genes (PCGs), 22 tRNA genes, two rRNA genes, an origin light-strand replication (Ori-L, a small DNA region between *tRNA^Asn^* and *tRNA^Cys^* genes that form a stem-loop structure, which some studies have shown to be essential to start mtDNA light strand replication. It was determined according to the sequence blast comparison with the proximal species), and a control region (D-Loop). Among all 37 genes, *ND6* and eight tRNA genes (*tRNA^Gln^*, *tRNA^Ala^*, *tRNA^Asn^*, *tRNA^Cys^*, *tRNA^Tyr^*, *tRNA^Ser(UCN)^*, *tRNA^Glu^*, and *tRNA^Pro^*) were encoded on the L-strand, the rest PCGs and tRNA genes were encoded on the H-strand (Figure 2). Twelve mitochondrial PCGs for *R. szechuanensis* shared the regular initiation ATG, only the cytochrome c oxidase 1 (*COX1*) gene began with GTG, as found in other gobies (Yang et al. 2020; Tan et al. 2020). There were four different patterns of termination codons: TAA, TAG, T, and TA. The conserved 13 PCGs varied from 168 bp (*ATP8*) to 1839 bp (*ND5*). The lengths of 22 tRNAs ranged from 67 to 76 nucleotides. The large ribosomal gene (16S) was 1639 bp and the small (12S) was 950 bp, which was divided by *tRNA^Val^.* The 839 bp-long control regions were identified between *tRNA^Phe^* and *tRNA^Pro^* (Table 1).

**Table 1.** Characteristics of the mitochondrial genome of *R. szechuanensis.*

Gene	Start	Stop	Length (bp)	Space (+) overlap (−)	Codons	
					initial terminal	Strand
*tRNA^Phe^*	1	68	68	0		H
*12S rRNA*	69	1018	950	0		H
*tRNA^Val^*	1019	1090	72	45		H
*16S rRNA*	1136	2774	1639	0		H
*tRNA^Leu^*	2775	2849	75	0		H
*ND1*	2850	3824	975	3	ATG TAA	H
*tRNA^Ile^*	3828	3897	70	−1		H
*tRNA^Gln^*	3897	3967	71	−1		L
*tRNA^Met^*	3967	4035	69	0		H
*ND2*	4036	5082	1047	1	ATG TAA	H
*tRNA^Trp^*	5084	5154	71	2		H
*tRNA^Ala^*	5157	5225	69	1		L
*tRNA^Asn^*	5227	5299	73	2		L
Ori-L	5302	5332	31	−1		H
*tRNA^Cys^*	5332	5398	67	0		L
*tRNA^Tyr^*	5399	5468	70	1		L
*COX1*	5470	7023	1554	0	GTG TAA	H
*tRNA^Ser^*	7024	7094	71	3		L
*tRNA^Asp^*	7098	7169	72	3		H
*COX2*	7173	7863	691	0	ATG T	H
*tRNA^Lys^*	7864	7939	76	1		H
*ATP8*	7941	8105	165	−7	ATG TAA	H
*ATP6*	8099	8781	683	0	ATG TAA	H
*COX3*	8782	9565	784	0	ATG TA	H
*tRNA^Gly^*	9566	9636	71	0		H
*ND3*	9637	9985	349	0	ATG TAG	H
*tRNA^Arg^*	9986	10054	69	0		H
*ND4L*	10055	10351	297	−7	ATG TAA	H
*ND4*	10345	11725	1381	0	ATG T	H
*tRNA^His^*	11726	11794	69	0		H
*tRNA^Ser^*	11795	11862	68	4		H
*tRNA^Leu^*	11867	11939	73	0		H
*ND5*	11940	13778	1839	−4	ATG TAA	H
*ND6*	13775	14296	522	0	ATG TAG	L
*tRNA^Glu^*	14297	14365	69	5		L
*CYTB*	14371	15511	1141	0	ATG T	H
*tRNA^Thr^*	15512	15583	72	−1		H
*tRNA^Pro^*	15583	15652	70	0		L
D-loop	15653	16491	839	0		H

### 3.2. Phylogenetic analysis

**Figure 3.**
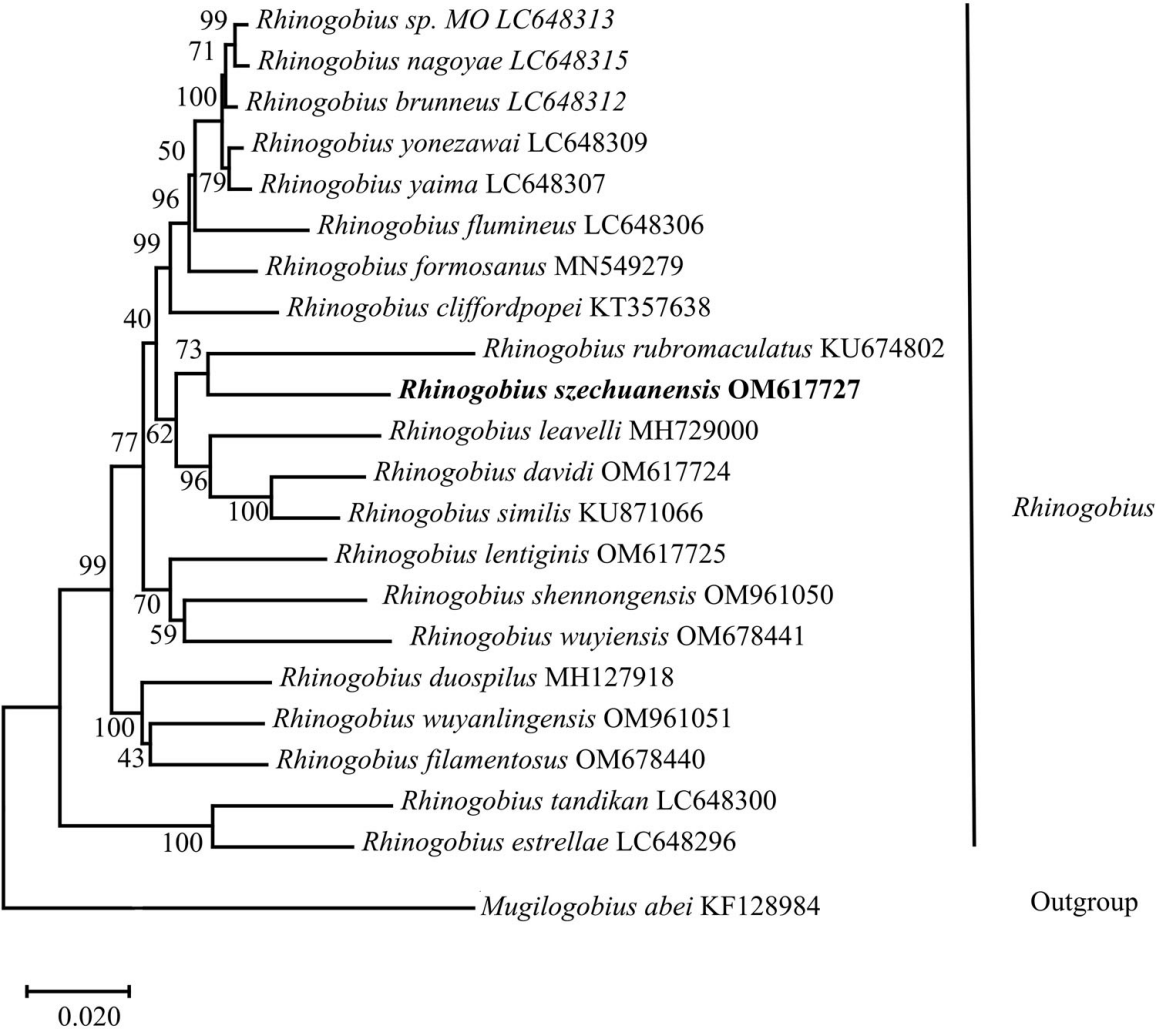
Maximum-likelihood (ML) phylogenetic tree was reconstructed based on the concatenated 13 protein-coding genes of *R. szechuanensis* and other 22 Gobionellinae fishes. Accession numbers were indicated after the species names. Numbers at the nodes indicated bootstrap support values from 1000 replicates.

The phylogenetic position of *R. szechuanensis* was inferred by a phylogenetic tree using MEGA X based on the combined 13 PCGs for 23 Gobionellinae species (Cheng et al. 2005; Huang et al. 2013; Huang et al. 2015; Xie et al. 2015; Wang et al. 2019; Zhang and Shen 2019; Tan et al. 2020; Yang et al. 2020; Maeda et al. 2021). As shown in Figure 3, the topological structure of the phylogenetic tree showed that *R. szechuanensis* and *R. rubromaculatus* gathered together, and they formed a sister group with the group include *R. leavelli*, *Rhinogobius davidi,* and *Rhinogobius similis.* The relationships among *Rhinogobius* were congruent with previous phylogenetic studies (Song et al. 2022). The results of phylogenetic analysis revealed the phylogenetic position of *R. szechuanensis* in the genus *Rhinogobius* at the molecular level. *R. szechuanensis* were clustered with all fish species of *Rhinogobius*, and within the genus *R. szechuanensis* and *R. rubromaculatus* were closely related.

## 4. Conclusions

We reported the first complete mitochondrial genome assembly and annotation of *R. szechuanensis* using the next-generation sequencing technology. The circular mitogenome was 16,492 bp in length, contained 37genes encoding 13 PCGs, 22 tRNAs and two rRNAs. The phylogenetic position was inferred by a Maximum-likelihood phylogenetic tree based on the concatenated amino acid sequences of 23 Gobionellinae species, which supported that *R. szechuanensis* was grouped together with *R. rubromaculatus*, and clustered with other *Rhinogobius* species. In brief, molecular data revealed that it should belong to *Rhinogobius,* which justified without doubt the current classification status as correct and valid. The mitochondrial genomic data of *R. szechuanensis* provided in this study will aid future research on evolution, taxonomy, DNA barcoding and population genetics of *Rhinogobius* species.

## Data Availability

The genome sequence data that support the findings of this study are openly available in GenBank of NCBI at (https://www.ncbi.nlm.nih.gov/) under the reference number OM617727. The associated “BioProject”, “Bio-Sample” and “SRA” numbers are PRJNA808189, SAMN26035968, and SRR18065517 respectively.
